# Species tropism of HIV-1 modulated by viral accessory proteins

**DOI:** 10.3389/fmicb.2012.00267

**Published:** 2012-07-26

**Authors:** Masako Nomaguchi, Naoya Doi, Yui Matsumoto, Yosuke Sakai, Sachi Fujiwara, Akio Adachi

**Affiliations:** Department of Microbiology, Institute of Health Biosciences, The University of Tokushima Graduate School,Tokushima, Japan

**Keywords:** HIV-1, species tropism, accessory protein, Vif, Vpu

## Abstract

Human immunodeficiency virus type 1 (HIV-1) is tropic and pathogenic only for humans, and does not replicate in macaque monkeys routinely used for experimental infections. This specially narrow host range (species tropism) has impeded much the progress of HIV-1/acquired immunodeficiency syndrome (AIDS) basic research. Extensive studies on the underlying mechanism have revealed that Vif, one of viral accessory proteins, is critical for the HIV-1 species tropism in addition to Gag-capsid protein. Another auxiliary protein Vpu also has been demonstrated to affect this HIV-1 property. In this review, we focus on functional interactions of these HIV-1 proteins and species specific-restriction factors. In addition, we describe an evolutional viewpoint that is relevant to the species tropism of HIV-1 controlled by the accessory proteins.

## INTRODUCTION

Human immunodeficiency virus type 1 (HIV-1) is strictly adapted to humans, and cause disease-inducing persistent infection only in humans (Nomaguchi et al., 2008). This property is unique among primate immunodeficiency viruses, and represent one of the most evident and important viral characteristics to understand the biology/molecular biology of HIV-1. Of numerous primate immunodeficiency viruses so far identified (Kirchhoff, 2009; Sharp and Hahn, 2011), HIV-1 with an extremely limited host range exhibits exceptionally high replication ability, transmissibility, and pathogenicity in sensitive host humans. For basic HIV-1 researchers, it would be final goal to realize the basis/mechanism underlying these properties by experimental approaches.

**FIGURE 1:**
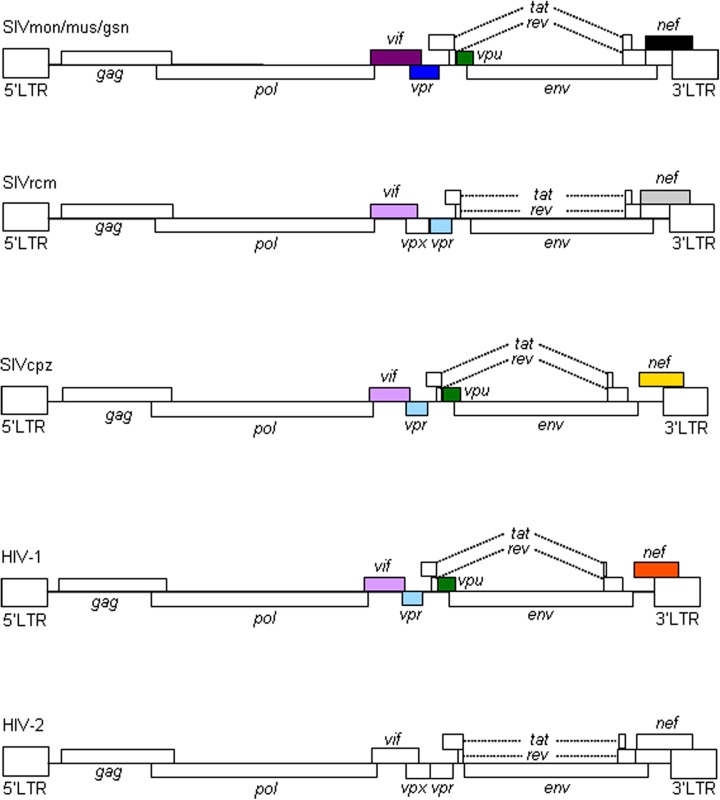
**Genome organization of primate immunodeficiency viruses.** Various proviral genomes are schematically shown. As indicated by colored boxes, the *vpr* and *vpu* genes of SIVcpz/HIV-1 came from those of SIVrcm and SIVmon/mus/gsn, respectively. Also, the* vif* genes of SIVcpz/HIV-1 originated from that of SIVrcm. In addition, as shown by colored boxes, HIV-1 *nef* gene is similar to but distinct from SIVcpz *nef* gene. HIV-1 *nef* gene is different from those of SIVmon/mus/gsn, SIVrcm, and HIV-2 as indicated. For virus designations, see text.

Primate immunodeficiency viruses can be divided into three groups based on their genome structure in the central regions (Kirchhoff, 2009; Fujita et al., 2010; Sharp and Hahn, 2011). While viruses of HIV-1 type contain *vpr* and *vpu* genes, viruses of HIV-2 type carry *vpx* and *vpr* genes in tandem (**Figure 1**). The other simian immunodeficiency viruses (SIVs), the prototype virus, have only the *vpr* gene in the corresponding genomic region. HIV-1 is believed to emerge from the prototype virus via SIVmon/mus/gsn (isolated from the mona, mustached, and greater spot-nosed monkeys), SIVcpz (isolated from the chimpanzees), and SIVgor (isolated from the gorilla) through mutational and recombinational events. SIVmon/mus/gsn is known to recombine with SIVrcm (isolated from the red-capped mangabey monkey) to generate SIVcpz (for genome structures, see, **Figure 1**). SIVcpz served as parental virus for HIV-1 (M and N) and SIVgor (and finally for HIV-1 P).

The biological and molecular biological bases for species tropism of HIV-1 should reside in the above outlined evolutional processes. In particular, the so-called accessory proteins encoded by extra genes are important. Each virus group has a unique set of the accessory proteins in terms of their combinations and of their activities. Therefore, studies on viral accessory proteins are also meaningful for understanding viral evolution by cross-species transmission.

## VIRAL AND CELLULAR DETERMINANTS FOR HIV-1 SPECIES TROPISM

Our early studies have already suggested the possible viral determinants and viral replication stage involved in the HIV-1 species tropism described above (Shibata et al., 1991, 1995; Shibata and Adachi, 1992). By the use of numerous chimeric molecular clones between HIV-1 and dual-tropic (tropic for human and monkey cells) SIVmac (isolated from the macaque monkey), we have claimed in essence, together with a work on the cyclophilin A (CypA; Dorfman and Gottlinger, 1996), that Gag-capsid (CA) and a viral protein(s) encoded by the central genomic region of HIV-1 are the determinants. We also have showed that HIV-1 is replication-incompetent in monkey cells because a certain replication step(s) before/during reverse transcription, other than the viral entry into cells, does not proceed normally. Subsequent extensive studies by us and others have clearly indicated that the interactions of Gag-CA/CypA, Gag-CA/tripartite motif (TRIM) proteins, and Vif/apolipoprotein B mRNA-editing enzyme-catalytic (APOBEC) proteins are major determinants for the HIV-1 species tropism (Nomaguchi et al., 2008, 2011; Nakayama and Shioda, 2012; Sakuma and Takeuchi, 2012) as summarized in **Table 1**. Gag-CA, CypA, and TRIM proteins have been described in detail in two articles in the Research Topic of this journal (Nakayama and Shioda, 2012; Sakuma and Takeuchi, 2012).

**Table 1:** Major viral and cellular determinants for HIV-1 species tropism.

Virus	Cell	Viral replication step affected
Gag-CA	CypA
Gag-CA	TRIM5α	Uncoating (early phase)
Gag-CA	TRIMCyp	Uncoating (early phase)
Vif	APOBEC3G	Reverse transcription (early phase)
	APOBEC3F	Reverse transcription (early phase)
Vpu	Tetherin/BST-2	Virion release (late phase)

*For details, see references (Nakayama and Shioda, 2012; Sakuma and Takeuchi, 2012) for Gag-CA, and Figures 3 and 4 for Vif/Vpu.*

## ACCESSORY PROTEINS OF PRIMATE IMMUNODEFICIENCY VIRUSES

**FIGURE 2:**
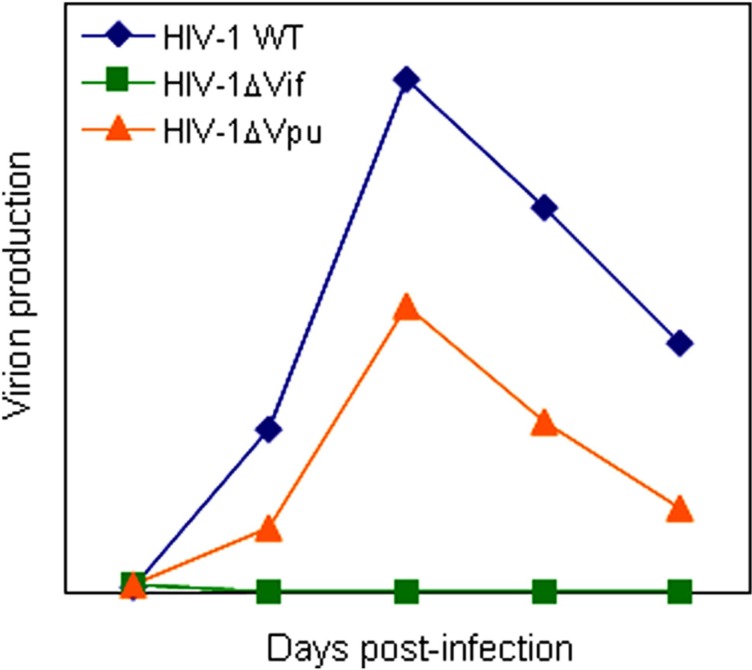
**A schema of replication kinetics by HIV-1 wild-type and mutant viruses.** Viral growth properties in cells are illustrated based on numerous infection experiments in our laboratory. WT, wild-type.

All primate immunodeficiency viruses encode a number of extra proteins (Vif, Vpx, Vpr, Vpu, and Nef) in addition to regulatory (Tat and Rev) and structural (Gag, Pol, and Env) proteins (**Figure 1**). Structural proteins are common to all retroviruses, but the regulatory and accessory proteins are unique to the complex primate lentiviruses and not found in the other simple mammalian retroviruses. Regulatory Tat and Rev proteins are trans-activators for transcription and for the expression of late viral proteins, respectively. While the regulatory and structural proteins are essential for viral replication, the extra proteins, unfairly generically called “accessory,” are dispensable under certain circumstances. However, in some cells, some of them are essential and the others are quite critical/important for optimal viral replication as illustrated for ΔVif and ΔVpu viruses (viruses that lack Vif or Vpu) in **Figure 2**. Another point to be mentioned here is relating to Vpr/Vpx proteins. Although Vpr and Vpx are genetically very similar (Khamsri et al., 2006), some primate immunodeficiency viruses bear two of them as described above (Fujita et al., 2010). Furthermore, the other viruses have Vpr only. What about the functional relationship of the two proteins? At present, the function of Vpr/Vpx is least well understood relative to that of the other accessory proteins (Malim and Emerman, 2008; Fujita et al., 2010). **Table 2** summarizes the important information regarding these accessory proteins so far reported. In total, it is fairly reasonable to believe that the accessory proteins are regulators to optimize viral replication and persistence *in vivo* thereby enhancing viral transmission between individuals.

**Table 2:** Accessory proteins of primate immunodeficiency viruses.

Viral Proteins	Major functions for viral replication reported so far
Vif	Neutralize APOBEC3G/F. Essential for viral replication in natural target cells.
Vpx	Degrade SAMHD1/APOBEC3A. Critical for viral replication in natural target cells.
Vpr	Important for viral replication in macrophages (HIV-1).
Vpu	Down-regulate Tetherin/BST-2. Important for viral replication in CD4-positive cells.
Nef	Down-regulate cell surface molecules (CD4, MHC-I etc.).

## Vif AND Vpu PROTEINS

**FIGURE 3:**
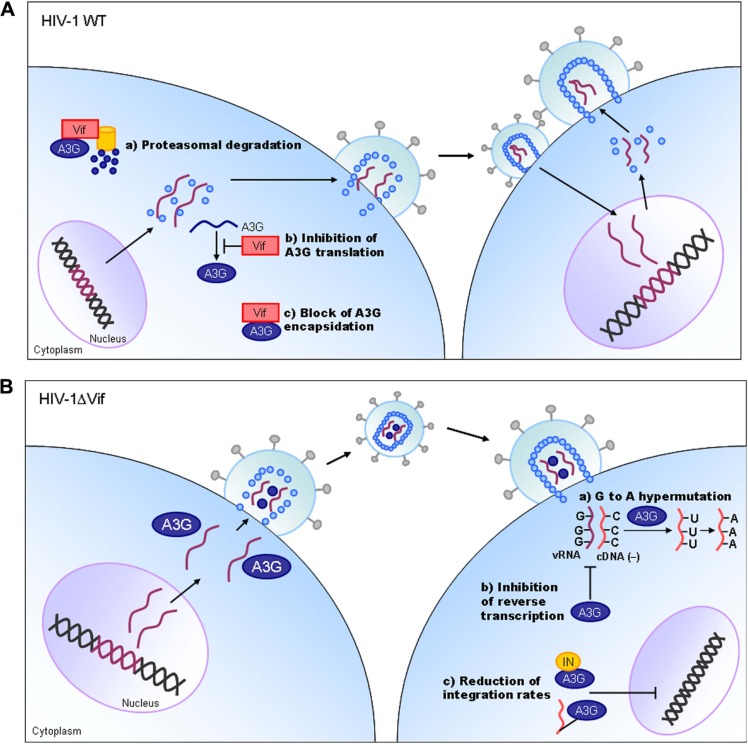
**HIV-1 replication and APOBEC3G.** On the basis of results reported so far, the action mechanism of Vif is depicted. Replication process for wild-type (WT) and ΔVif mutant viruses are schematically shown on the basis of previously reported review articles (Holmes et al., 2007; Huthoff and Towers, 2008; Strebel et al., 2009). A3G, APOBEC3G; IN, viral integrase protein.

Vif protein is essential for viral replication in natural target cells such as CD4-positve lymphocytes and macrophages. Recent identification of its cellular object for attack (Sheehy et al., 2002) has clearly revealed the biological and biochemical bases for the growth property of ΔVif virus in natural target cells. This finding (identification of a family of APOBEC3 proteins, cellular cytidine deaminases, as potent inhibitors of HIV-1 replication in primary cells) has also contributed much to establish the concept of “the restriction factor” to well understand virus–cell interaction (Malim and Emerman, 2008; Sato et al., 2012). Of the APOBEC3 family proteins, APOBEC3G and APOBEC3F (Kitamura et al., 2011) strongly inhibit viral replication in the absence of Vif (**Figure 3**). Although HIV-1 Vif can abrogate the activities of human APOBEC3, it cannot do so against monkey APOBEC3. In contrast, SIVmac Vif can neutralize the anti-viral activity of APOBEC3 of both origins. Finally, it has been demonstrated that Vif and APOBEC3 are the major determinants for the HIV-1 species tropism by constructing macaque-tropic HIV-1 (HIV-1mt) and monitoring the HIV-1mt growth property in various genetic contexts of macaques (Hatziioannou et al., 2006, 2009; Kamada et al., 2006; Igarashi et al., 2007; Thippeshappa et al., 2011).

**FIGURE 4:**
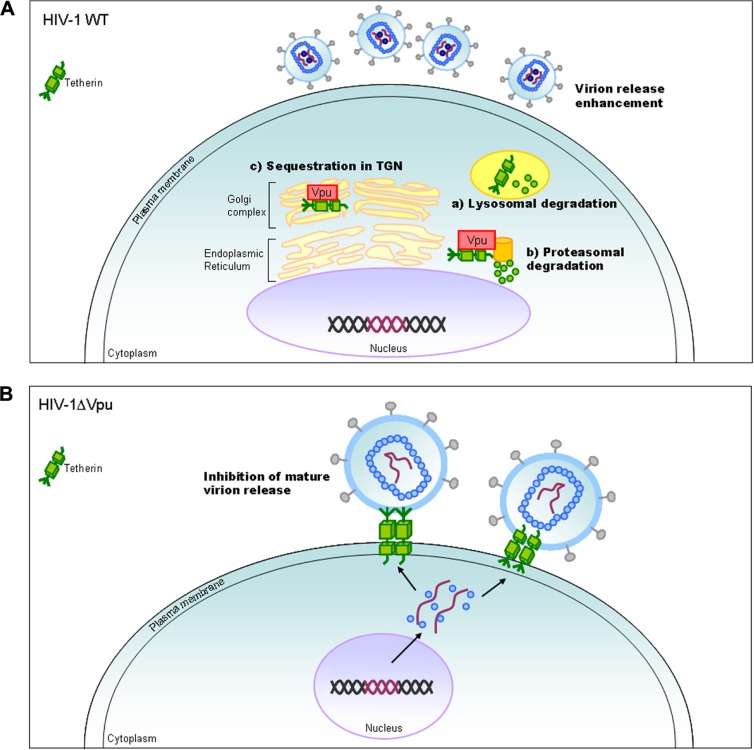
**HIV-1 replication and Tetherin.** On the basis of results reported so far, the action mechanism of Vpu is depicted. Replication process for wild-type (WT) and ΔVpu mutant viruses are schematically shown on the basis of previously reported review articles (Tokarev et al., 2009; Douglas et al., 2010; Evans et al., 2010). TGN, trans-Golgi network.

Vpu protein, unique to viruses of the HIV-1 group (**Figure 1**), modulates viral replication in human CD4-positive cell lines and primary cells. Mutant HIV-1 without Vpu (ΔVpu virus) grows poorly relative to wild-type virus. Recently, a cellular protein named Tetherin (also called BST-2) has been identified as a restriction factor against HIV-1 and is antagonized by Vpu (Neil et al., 2008; Van Damme et al., 2008). Vpu down-regulates the Tetherin from cell surface, and thereby promotes extracellular production of progeny virions (Malim and Emerman, 2008; Arias et al., 2011; Sato et al., 2012). The baseline mechanism for this action of Vpu is well studied as shown in **Figure 4**. Here, it must be attentive that the anti-Tetherin activity of Vpu is host species-specific as observed for Vif. HIV-1 Vpu acts against human but not (or poorly) macaque Tetherins (Sauter et al., 2009, 2010). Although the biological effect of Vpu is much milder than that of Vif as judged by the growth kinetics of mutant viruses (**Figure 2**), Vpu may be critical for interspecies transmission through mutation/adaptation/recombinations (Kirchhoff, 2009; Sauter et al., 2009, 2010; Sharp and Hahn, 2011). Thus, Vpu and Tetherin affect the HIV-1 species tropism, but the effect may be relatively small.

In sum, Vif and Vpu counteract the major restriction factors APOBEC3 proteins and Tetherin/BST-2, respectively, and represent viral determinants for the host range of HIV-1 (**Tables 1 and 2**). It is intriguing to note that these factors would have shaped HIV-1 and made it unique among various primate immunodeficiency viruses (**Figure 1**).

## Vpx AND Vpr PROTEINS

Vpx and Vpr proteins are necessary for efficient viral replication (Malim and Emerman, 2008; Fujita et al., 2010). In macrophages, ΔVpx replication is not detectable and this defect has been shown to be present at post-entry and before/during the reverse transcription process (Fujita et al., 2008, 2010; Srivastava et al., 2008). Also in some lymphocyte cell lines and in primary lymphocytes, Vpx protein is critical for viral replication (Ueno et al., 2003; Fujita et al., 2010; Doi et al., 2011). Because ΔVpr virus is somewhat replication-defective in some cells (for both HIV-1 and HIV-2), it is not unreasonable to assume that Vpr may play a role in the viral growth cycle. As such, Vpx and Vpr are important for *in vivo* viral replication and finally for viral pathogenicity (Fujita et al., 2010).

Very recently, SAMHD1 and APOBEC3A have been reported to be myeloid cell-specific restriction factors against HIV-1 counteracted by Vpx (Berger et al., 2011; Hrecka et al., 2011; Laguette et al., 2011). Whether these proteins are associated with the HIV-1 species tropism described in this review article, and whether they can explain the *in vitro* and *in vivo* situation of HIV-2/SIVmac mutant viruses mentioned above remain to be determined (Fujita et al., 2010; Nomaguchi et al., 2011).

## CONCLUSION

In this review, we have described the major determinants for the species tropism of HIV-1. Structural Gag-CA and accessory Vif and Vpu proteins are clearly involved in this host range of HIV-1 as viral factors (**Table 1**). Cellular proteins that interact with these and contribute to this tropism are definitely the restriction factors (**Table 1**). In total, interplays between the viral and cellular responsible factors decide this unique and limited tropism of HIV-1. Whether there are the other factors that affect the HIV-1 species tropism is awaiting further investigations. In this regard, the biology of Vpx deserves attention. Because Vpx is present in SIVmac but not in HIV-1 (**Figure 1**), it may inactivate a cellular anti-viral protein(s) which is not recognized by HIV-1 proteins.

In both basic and applicable points of view, the narrow host range of HIV-1 is burdensome obstacle to overcome. Assuming that HIV-1mt can grow and cause disease similarly with SIVmac in macaques, we would be able to better perform model studies to precisely analyze viral replication and pathogenicity *in vivo*, and to establish the effective anti-HIV-1/AIDS strategies. To the best of our knowledge, there are no such HIV-1mt clones so far (Hatziioannou et al., 2006, 2009; Kamada et al., 2006; Igarashi et al., 2007; Kuroishi et al., 2009; Saito et al., 2011; Thippeshappa et al., 2011). We may further improve the ability of HIV-1mt by today’s powerful methodology if we knew all the cellular determinants for the species tropism of HIV-1. Studies in this direction are in progress in our laboratory.

## Conflict of Interest Statement

The authors declare that the research was conducted in the absence of any commercial or financial relationships that could be construed as a potential conflict of interest.
